# Overview of Traditional Mongolian Medical Warm Acupuncture

**DOI:** 10.14336/AD.2022.0115

**Published:** 2022-07-11

**Authors:** Guo Shao, Wei Xie, Xiaoe Jia, Rengui Bade, Yabing Xie, Ruifang Qi, Kerui Gong, Haihua Bai, Lengge Si, Yingsong Chen, Kai Sun, Agula Bo

**Affiliations:** ^1^Center for Translational Medicine and Department of Laboratory Medicine, the Third People’s Hospital of Longgang District Shenzhen, Shenzhen, China.; ^2^Inner Mongolia Key Laboratory of Hypoxic Translational Medicine, Baotou Medical College of Neuroscience Institute, Baotou Medical College, Baotou, China.; ^3^Beijing Key Laboratory of Hypoxic Conditioning Translational Medicine, Xuanwu Hospital, Capital Medical University, Beijing, China.; ^4^Department of Oral and Maxillofacial Surgery, University of California San Francisco, San Francisco, USA.; ^5^Inner Mongolia Minzu University, Tongliao, China.

**Keywords:** Mongolian medicine, warm acupuncture, age-related diseases

## Abstract

Mongolian medical warm acupuncture is a traditional therapy of Mongolian medicine and was developed by people living on the Mongolian Plateau. This kind of traditional oriental medicine has a long history. The main characteristics of Mongolian medical warm acupuncture are the acupoints and the needles used. Its theory is based on the human anatomical structure and the distinct local culture. Mongolian medical warm acupuncture has been practiced for centuries and proved to be very effective in the treatment of age-related diseases, including the musculoskeletal and nervous diseases. This paper aims to briefly introduce the history and scope of Mongolian medical warm acupuncture, with a particular focus on age-related diseases, where Mongolian medical warm acupuncture has shown significant beneficial effects.

Traditional Chinese medicine (TCM) is one of the oldest and most unique medical systems in the world with a written history of nearly 3,000 years [1]. TCM is still practiced throughout China and has been subject to modifications and further developments. Traditional Mongolian medicine (TMM), similar to TCM, is still practiced in Mongolia and the Inner Mongolia Autonomous Region of China [2]. Because traditional medicines from different areas may have a common Chinese origin, traditional oriental medicine (TOM) has been declared by the World Health Organization (WHO), and more than 3,000 basic nomenclatures have been used to construct a database for the retrieval of various published scientific articles [3]. Research on TMM may contribute to the development of TOM.

Acupuncture is a component of TOM and a traditional therapy used in the Orient to treat disorders of internal viscera through stimulation of the body surface by silver needles [4]. Mongolian acupuncture is a therapy with a long history. A stone needle of Neolithic age was discovered in the Xiligol League of Inner Mongolia in the 1960s, bronze needles were discovered in the Spring and Autumn Period, and the Warring States Period was discovered in the Ikezhao League of Inner Mongolia in the 1970s [5]. Moxibustion is well known to be a traditional Mongolian therapy. The *“Yellow Emperor's Classic of Internal Medicine”*states that the origin of warm acupuncture is “North.” We can infer that the “North” region refers to the Mongolian Plateau because of the descriptions of nomadic living and the cold climate. Mongolian moxibustion was recorded in “ *Four Medical Tantras”* written by YutuoYundan-Kampot, a famous physician of the 8^th^ century AD. Therefore, Mongolian warm acupuncture may have been employed by people living on the Mongolian Plateau for centuries [6].

The use of warm acupuncture to treat disease depends on the culture of Mongolian people. People living on the Mongolian Plateau have achieved an understanding of the human anatomical structure through celestial burials and hunting. They also adopted medical practices from TCM, most prominently implementing the Yin and Yang theory for their diagnoses and treatments. They combined their anatomic knowledge and Tibetan Buddhism with TCM theories to create a medical system that has unique cultural features [2]. The TMM theory is based on the idea that the “Black Meridian” and “White Meridian” systems connect the body with the surrounding environment; they control the exchange of matter and information between the inside and the outside. Currently, the “Black Meridian” and “White Meridian” systems are regarded as the circulation and neural systems, respectively, in the modified TMM theory [7]. In TMM, the “White Meridian” system comprises the brain and spinal cord, and includes the inner and outer “White Meridian”[8]. The inner “White Meridian” controls internal organs (heart, small intestine, lungs, colon, liver, gallbladder, spleen, stomach, kidneys, and bladder), thus its function is similar to that of the autonomic nervous system.

Acupuncture is a set of medical treatments and an ideology based on the principle of applying small needles or pressure to specific points, presenting a dermal projection of the body’s internal viscera [9]. In the TMM system, there are certain special points (acupoints) to which warm needles may be applied to treat different diseases. Some models were set up for Mongolian physicians to practice the identification of acupoints [10]. There are more than 42 acupoints on the neck and more than 150 on the trunk, with most of these acupoints on the “White Meridian” (Fig. 1).

**Figure 1.**
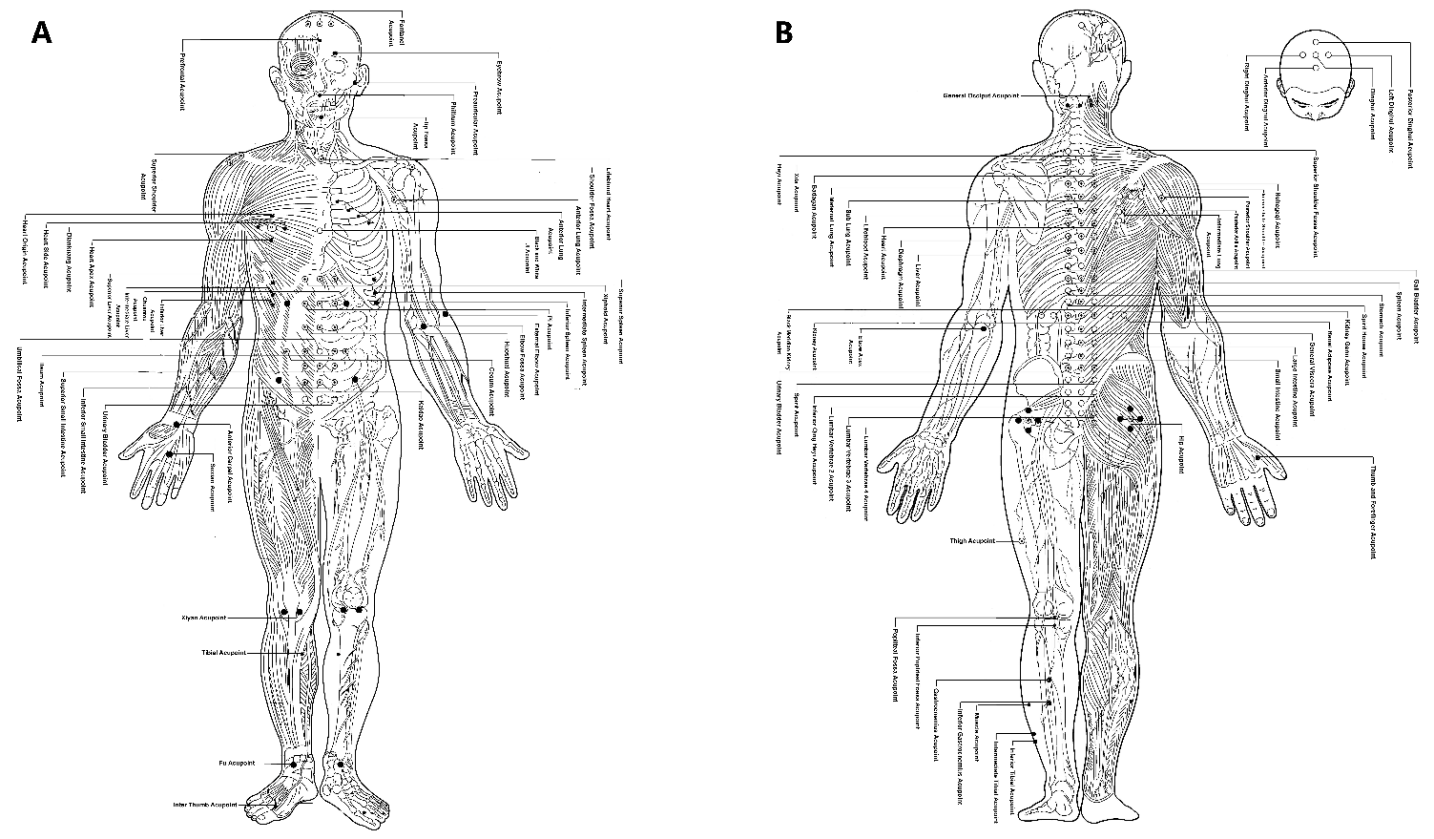
**The map of Mongolian medicine acupoints**. (**A**) Anterior view. (**B**) Posterior view.

Acupoints in TMM are those points that can balance the internal environment and regulate temperature when stimulated. The selection of acupoints is based on dialectical treatment, which uses the etiology and pathological changes for theoretical guidance. These factors, including the “Black Meridian” and “White Meridian,” pain location, and diseased areas are to be considered in the choice of the acupoints. For example, the Dinghui acupoint mainly treats symptoms such as those of the Heyi fever, which causes dumbness, confusion, and memory loss, based on the records of the “ *Encyclopedia of Mongolian Studies (Medical).*” Stimulation of the Heyi acupoint can be used to treat dizziness and headaches, including those caused by disease. The basic principle for acupoint choice is maximizing the effects with fewer points. The selection of the acupoints on behalf of the doctors is based on recorded documentation or their own experience.

During the past 3,000 years, many cases of various diseases have been treated by acupuncture in Asian countries. These diseases are often considered to be neurovegetative disorders. Mongolian warm acupuncture has diverse effects on the body: (1) it can reduce inflammation and promote the reabsorption of chronic inflammation; (2) it may have analgesic effects and raise the pain threshold; and (3) can have a regulatory effect on the immune system [11]. Mongolian warm acupuncture was effective in treating certain diseases related with aging, such as bone [12], neuronal [13], and metabolic diseases.

## Basic principles of Mongolian medicine and its anti-aging effects

Mongolian medicine is a type of TOM. Its characteristics are based on the experience accumulated by the Mongolian ancestors in their long-term life practice, while absorbing the essence of other (Chinese medicine, Tibetan medicine) ethnic and ancient Indian medicines. TMM is guided by simple materialism and spontaneous dialectics. A unique ethnic medicine gradually formed and developed. The basic theories of Mongolian medicine are based on experience from clinical practice. These theories include those of Yin and Yang, the five Yuan (earth, water, fire, air, empty) and the five elements (gold, wood, water, fire, earth), the three roots (Heyi, Shila, Badagan) and seven Su (food, blood, muscle, fat, bone, bone marrow, energy), among others. The three roots and seven Su are the most important theories in Mongolian medicine. The three roots control different parts of the body (Heyi and Badagan each control the upper and lower body, respectively above the heart and under the navel, while Shila controls the trunk, between the heart and navel). The seven Su are the materials that build the body. The three roots and seven Su are the foundation of the Mongolian medicine theory. A disorder in these elements is considered the reason for disease in TMM [14].

The three methods of diagnosis generally adopted by TMM doctors are the basic methods for illness diagnosis: observation, inquiry, and palpation. These basic methods are very similar to the four methods of diagnosis used in TCM. There are four treatment methods used in TMM, namely oral medicine (such as herb), therapeutic techniques (such as acupuncture and bloodletting), beverages and food (such as yogurt made with horse milk), and movement (such as Andai dancing). Mongolian warm acupuncture is part of the therapeutic techniques of the Mongolian medicine system. Traditional Chinese acupuncture has been shown to be useful in clinical therapy and to possess anti-aging properties [15]. The imbalance of the three roots was regarded as the reason for aging. It has been demonstrated that six Mongolian acupoints, including Heyi, Dinghui, and Huoshuai, have stable anti-aging effects in Mongolian warm acupuncture [16].

## History of Mongolian acupuncture and warm acupuncture

Mongolian ancestors invented acupuncture therapy. Acupuncture therapy belongs to TMM, has a long history and unique curative effects. Mongolian acupuncture can conduct heat or cold and produce stimulation at fixed acupoints on the human body. Archaeology has proven, in recent years, that Mongolian ancestors made and used special tools for traditional medicine as early as 6,000 years ago, in the Neolithic age or even earlier [17]. Medical stone needles, bone needles, bronze flint needles and other cultural relics unearthed in Inner Mongolia all explain the origin of acupuncture and moxibustion [18], showing that the ancestors living in the Xiligol and the Ikezhao Leagues of Inner Mongolia had mastered these techniques [19]. From the 11^th^ to the 3^rd^ century B.C., people already knew how to treat certain diseases with acupuncture and bloodletting therapies. Between the 17^th^ and 18^th^century, the famous Mongolian physician Yixibalajur wrote the first record of indications, contraindications, efficacy, and acupoints of Mongolian acupuncture, which laid the foundation for the widespread use of acupuncture in clinical practice. *“Four Medical Tantras · Blue Beryl”* quotes that *“acupuncture is to correct the five kinds of surgical treatment errors.”*The *“Encyclopedia of Chinese Medicine · Mongolian Medicine”* reports the use of iron, bone, bronze, copper, silver, and gold needles in the process of development of Mongolian medicine acupuncture. Among these needles, the silver needles are mainly used [20]. The silver needle has a strong bactericidal effect, does not deteriorate over long periods of time, presents appropriate hardness and good elasticity, and is therefore widely used in the clinical practice.

Due to the late definition of Mongolian characters, there are only scattered fragments of records in literature regarding the ancient medical activities of Mongolians and the process of development of acupuncture therapy. Ancient books written in Chinese and Tibetan in the Zhanguo and Qin/Han Dynasties contained records of Mongolian medicine and medical treatment, but the content of such records was relatively fragmented and did not form a theoretical system. The prescriptions and treatments, such as Mongolian moxibustion and Xiongnu sleeping pills commonly used by northern people, are recorded in the *“Four Medical Tantras”* and *“Bei Ji Qian Jin Yao Fang: Essential Prescriptions worth a Thousand in Gold for Every Emergency.”* In the 13^th^ century, *“The Secret History of the Mongol”* reports that, before the Yuan Dynasty, Mongolians used kefir to treat injured patients with bleeding and fainting. Because of the Indian medical book *“Ashtanga Hridya Samhita”* and Tibetan medicine *“Four Medical Tantras*, *”* the theoretical system of Mongolian medicine improved and further developed. Many Mongolian medical scientists emerged, and medical works about Mongolian medicine written in Tibetan or Mongolian were published. The monograph *“Zhu Shi Chu Hei Ming Deng”* edited by Darimaomaremba Lobsang Asahiga reports content related to the acupuncture techniques of Mongolian medicine from different perspectives and has had a great influence on the development of Mongolian medicine acupuncture techniques in later generations. Mongolian acupuncture therapy, guided by the basic theories of Mongolian medicine and based on syndrome differentiation, uses special needles to penetrate specific parts of the human body to stimulate and regulate the three roots, and dredge the “White Meridian,” achieving a traditional therapy for preventing and curing diseases.

Before the 1950s, warm acupuncture was only one of the TMM techniques in Mongolian medicine. The existence of warm acupuncture was based on the geographical environment and living habits of a nomadic people. Moxibustion and acupuncture in Mongolian medicine have a long history. The development of warm acupuncture in Mongolian medicine can be divided into an accumulation period (before the 1950s), a formation period (between the 1950s and the end of the 20^th^ century), and a prosperity period (from the 21^st^ century to the present). The name of “warm acupuncture” is not ancient but gradually became clear with the development of Mongolian medicine. The evolution of the name has gone through different phases: hot needling plus fire moxibustion, hot needle assisting heat, warm moxibustion needle, warm needle, warm needling, and finally warm acupuncture. Mongolian medicine warm acupuncture was listed on the national intangible cultural heritage list, which allowed it to have a formal identity. As an invented tradition, the Zanbula Dorje warm acupuncture aims to create the identity of “national medicine - traditional status.”

**Figure 2.**
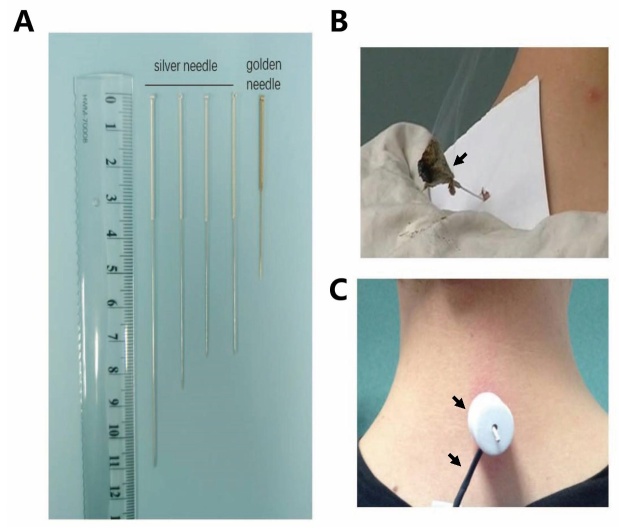
**Illustration of Mongolian acupuncture needle and Mongolian warm acupuncture**. (**A**) The acupuncture needles commonly used in Mongolian acupuncture. (**B**) The representative image showing Traditional Mongolian warm needles, which heated by burned moxa. The arrow indicated that the burned moxa was heating up the needle. (**C**) The representative image showing Modern Mongolian warm needle, which heated by electronically. Tthe arrows indicated that the electric heating unit was heating up the needle.

Modern needles used in Mongolian warm acupuncture have been continuously modified with the advancement of science and technology. Modern Mongolian “warm acupuncture” is used safely and effectively in hospitals. The needle is heated electronically (Fig. 2C) and the diameter of the silver needle has been reduced. The method for temperature control has been improved, and resistance heating is used to replace mugwort burning. The new heating technology can not only achieve the thermal efficiency of traditional silver needle heating, but is also easy to operate and smoke-free, overcoming the issue of moxa grass smoke [21]. These thin silver needles can reduce damage as effectively as those used in traditional warm acupuncture.

## Types of Mongolian acupuncture and warm acupuncture

Warm acupuncture is a needle method where the tail of a filiform needle is wrapped with moxa, the combustion of which generates heat to cure diseases. Warm acupuncture combines the effects of acupuncture and moxibustion; the needles are passed through the body acupoints to warm the meridians and promote the circulation of Qi and blood. Warm acupuncture is used to treat cold stagnation in the meridians, Qi and blood blockage, and other diseases [22]. With the increase in acupuncture research, a variety of needles have emerged. In *“Modern Acupuncture Equipment and Special Therapy*, *”* nine ancient, nine new, and various modern needles are systematically classified according to the purpose, shape of the needle handle, material of the needle body, and specifications [23]. The needles used in Mongolian medicine acupuncture are generally silver or gold. In the development process, bone, iron, bronze, copper, silver, and gold needles were used (Fig. 2A). There were eight types of Mongolian acupuncture needles recorded in the *“Gan Lu Si Bu,”* and these types were divided into three types with holes and five without. Mongolian medicine silver needles are widely used clinically because they do not rust easily, are hard but present good elasticity, have strong thermal conductivity and are suitable for warm acupuncture [24-28]. In the research *“Origin of Acupuncture Technique*, *”* the opinions of various experts are also divided. TCM acupuncture techniques were first recorded in the *“Yellow Emperor's Classic of Internal Medicine*. *”* The theory of acupuncture techniques, symptoms, acupunctures and moxibustion methods are also described in the *“Yellow Emperor's Classic of Internal Medicine*. *”* Especially in *“Ling Shu*, *”* significant space is devoted to acupuncture techniques. In the Wei and Jin Dynasties, Huang Fu Mi (215-282)[29] systematically explained acupuncture and moxibustion and compiled the *“A-B Classic of Acupuncture and Moxibustion*. *”* This valuable text is the earliest existing monograph on acupuncture and moxibustion integrating theory with practice. Known as the ancestor of TCM acupuncture, it provides specific guidance and a theoretical basis for clinical treatments performed by acupuncturists. Modern TCM acupuncture techniques employ ancient traditional acupuncture, enriched, and perfected by the combination with modern science and technology.

Mongolian medicine acupuncture is divided into hot and cold, with hot acupuncture comprehending fire needle, hot moxibustion combined with acupuncture, and hot moxibustion combined with fire needle, while cold acupuncture includes pure acupuncture, warm acupuncture, and cold acupuncture. Since the 16^th^ century, Mongolian medicine scholars have paid increasing attention to the theory and operation of warm acupuncture. In the book *“Gan Lu Si Bu”* we find written that *“acupuncture has two kinds of cold needling amounts and hot acupuncture*, *”* explaining that “cold acupuncture” is acupuncture alone, while “hot acupuncture” is fire acupuncture deriving from a combination of acupuncture and combustion. Among these types of acupuncture, warm acupuncture has a long history. Warm acupuncture is based on the basic theory of Mongolian medicine and the treatment principle of TMM. It uses a special needle to heat the body surface through fixed acupoints so that warm stimulation can be transmitted into the body through the needle to treat diseases, also known as “turimu” [30,31].

Developed in modern Mongolian medicine, the therapeutic use of acupuncture is recorded in detail in the *“Mongolian Medicine Clinic”*edited by Ce Su Rongzhabu. This book has a high theoretical and practical clinical value. Warm needling therapy is a type of acupuncture in both TCM and TMM, first seen in TCM in the *“Shang Han Lun Treatise on Febrile Diseases Caused by Cold”* by Zhang Zhongjing in the Eastern Han Dynasty. The method used fragrant *Angelica dahurica* as round cake and moxibustion on the trocar to obtain the effect on the acupoints. A moxa rod was placed at the end of the needle, and, after the rod was ignited, the heat was transmitted to the body through the needle to prevent diseases. Warm acupuncture was regarded as a method capable of gathering and regulating Qi. Therefore, this method is still widely applied to treat diseases related to Qi.

In addition to the different acupuncture methods in TCM, warm acupuncture therapy, the forms of moxa used, and the methods of application in TMM warm acupuncture are also different. It can be divided into moxa moxibustion and moxibustion moxibustion [22]. Moxibustion takes advantage of the mild heat of moxibustion fire and the pharmacological effects of mugwort leaves through the meridian conduction. This method can warm the blood, strengthen the body, and eliminate disease, achieving its curative purpose. The Mongolian warm acupuncture is an excellent treatment with national and regional characteristics that the Mongolian people have developed through thousands of years. In accordance with the living environment and lifestyle, the Mongolian people have created warm acupuncture to treat diseases related to the cold temperatures. Mongolian warm acupuncture therapy is a combination of acupuncture and warm moxibustion and maintains the functions of both techniques. Mongolian medicine moxibustion is a treatment consisting in a needle, made of gold or silver, pierced into the target acupoint, and the needle handle is heated by moxa combustion or candle fire.

Through the inheritance and development of Mongolian medicine over several generations, the warm needle of Mongolian medicine has become an important treatment in contemporary Mongolian rehabilitation medicine. *“Gan Lu Zhi Quan, ”* part of the *“Gan Lu Si Bu, ”* is the earliest record of the types of acupuncture techniques, correction of acupuncture errors, acupuncture methods, acupoints, contraindications, and efficacy, among others [32]. Mongolian warm acupuncture has also undergone continuous development and innovation through the improvement and transformation of traditional techniques. Consequently, the therapeutic effect of Mongolian warm acupuncture has been reinforced. The method has been progressively upgraded from the original Mongolian warm moxa acupuncture to the current 1^st^ and 2^nd^ generation methods. At present, modern Mongolian medicine warm acupuncture therapy devices can easily control acupuncture temperature according to the different patients and their condition [33-36]. However, both TCM warm acupuncture and Mongolian warm acupuncture are adapted to the requirements of current times and the standardization of scientific research. Both are based on the idea that “innovation is not separated from the source” and seek common ground while keeping their differences.

## Effects of Mongolian warm acupuncture on age-related diseases

### Bone diseases

Degenerative osteoarthrosis, which includes gonarthrosis, cervical spondylosis (CS), and lumbar disc herniation (LDH), is a degenerative disease characterized by degenerative injury of articular cartilage and reactive hyperplasia of the articular margin and subchondral bone [37]. Clinical manifestations are mainly joint pain, tenderness, stiffness, joint swelling, limited movement derived from aging, obesity, strain, and trauma [38]. This disease often causes great pain and seriously affects the quality of life of patients. However, to date, there is still no effective drug to prevent disease progression, but only for alleviating pain [39]. However, Mongolian medicine has unique advantages and rich experience in the treatment of degenerative osteoarthrosis [40]. In particular, Mongolian warm acupuncture, as a safe and noninvasive therapy, has been widely used in the clinical treatment of degenerative osteoarthrosis and has achieved significant therapeutic effects [41].

According to TCM, LDH is caused by liver and kidney deficiencies, Qi stagnation, blood stasis, and external pathogenic factors [42].Warm needle moxibustion, as one of the treatment methods in TCM, has been reported to have a better therapeutic effect in relieving lumbago and lumbar dysfunction, and can upregulate blood β-EP levels [43]. However, LDH is considered to depend on the imbalance between Heyi, Xila, and Badagan at the TMM acupoint. The Qi and Xieriwu factors are concentrated in the lumbar joint, surrounding tissue, muscle, and fascia, resulting in the obstruction of the White Meridian function. Because of this, LDH is regarded as a “White Meridian” disease [44]. At present, some studies have found that Mongolian warm acupuncture can effectively treat LDH. In a clinical experiment, warm acupuncture was used to treat 73 cases of LDH by puncturing three spinal and Badagan acupoints. After 20 days of treatment (once daily), the overall effectiveness rate was 94.59% in all patients. The VAS score and IL-1β and TNF-α levels in the serum of patients were significantly lower than before treatment [45].In addition, other studies also showed that Mongolian warm acupuncture might improve the curative effect on LDH in a short period of time and reduce the degree of pain in patients [46].

Knee osteoarthritis (KOA) has been included in the category of “GuBi” in TCM. Due to aging or working for extended periods of time, KOA often stems from liver and kidney deficiencies, Yang deficiency, and blood stasis [47]. Four interventions of TCM, acupuncture, moxibustion, herbs, and massage may alleviate symptoms such as pain, swelling, and dysfunction in KOA [48]. Furthermore, TMM has also been studied in KOA for many years and is believed to be a Xieriwu factor disease. On one hand, the obstruction of Qi and blood circulation, the siltation of diseased blood, and Xieriwu factors may induce joint swelling or articular effusion. On the other, a reduction in joint lubricating fluid and joint cavity stenosis may occur due to the decrease in blood supply and Badagan [49]. Recent studies have shown that Mongolian warm acupuncture might be an effective method to treat KOA by puncturing the Xiyan acupoints (EX-LE5), Jingneice acupoints, and Qiangshen acupoints. For example, in a clinical experiment involving 1,500 KOA patients, the overall effective can reach 98.6% after one month of continuous treatment with Mongolian medicine warm acupuncture, and the VAS score of patients was significantly decreased compared with before treatment [50]. Moreover, compared with certain physical therapies, Mongolian warm acupuncture might quickly relieve pain and improve joint discomfort caused by KOA [51].

CS often results in neck pain. Nevertheless, four weeks of optimized acupuncture treatment, which is an important part of TCM, can alleviate CS-related neck pain [52]. There are two types of CS in TMM: cervical spondylotic radiculopathy and CS of the vertebral artery type. Between these, cervical spondylotic radiculopathy has the highest incidence. CS is caused by damage to the “White Meridian” of the head and neck resulting from the disorder of the three roots (Heyi, Xila and Badagan), which eventually leads to the loss of physiological functions. Therefore, CS belongs to the head and neck “White Meridian” disease group [53]. Mongolian medicine warm acupuncture can conduct heat stimulation to the diseased area and dredge the “White Meridian,” playing a therapeutic role in the treatment of cervical spondylotic radiculopathy [54]. A series of clinical experimental results also supports these effects, where 170 cases of CS were treated with Mongolian medicine warm acupuncture by puncturing the Dazhui, Fengfu, Tianzhu, Fengmen, Fengchi, and Jianjing points. Following up to seven treatments (one time every two days), Mongolian warm acupuncture might effectively relieve neck spasms, exert analgesic effects, and improve microcirculation functions [55].

Although Mongolian warm acupuncture is an effective method to treat degenerative osteoarthrosis, the detailed mechanism remains to be investigated. Some studies have speculated that the temperature of the tip of the needle in Mongolian warm acupuncture is 39-41 °C. This heat may penetrate through the muscle and be transmitted to the pathological tissue around the bone and joint, causing edema and atrophic degeneration of skeletal muscle cells to disappear [56]. In addition, Mongolian warm acupuncture may also destroy the collagen protruding outside the vertebral nucleus pulposus, thus alleviating the pressure of induced by the LDH protrusions on the nerve root and dural sac [57]. Self-repair of articular cartilage and reduction of joint friction may be activated by Mongolian warm acupuncture to protect the knee joint cartilage and articular surface in KOA patients [50]. Studies have indicated that Mongolian warm acupuncture may also suppress ischemia and inflammatory stimulation by promoting local blood circulation and down-regulating the expression of inflammatory factors (such as IL-1β, IL-6,TNF-α, IFN-γ) and iNOS [58], relieving pain by inhibiting the apoptosis of nucleus pulposus cells, or accelerating the release of analgesic substances such as the morphine peptide [59,60]. In particular, Mongolian warm acupuncture needles are generally made of silver, which plays an anti-inflammatory and bacteriostatic role [61]. In brief, more research on the mechanisms underlying the effects of Mongolian medicine warm acupuncture should be performed to advance its clinical application.

### Nervous system diseases

#### Effects on insomnia

Insomnia is a common disease caused by various factors, such as difficulty falling asleep, short sleep depth or frequency, early awakening, insufficient sleep time, or poor quality sleep [62]. Insomnia has become one of the most common diseases in neurological clinics, according to recent studies. Mongolian warm acupuncture may improve sleep quality and has few toxic side effects compared to hypnotic medications.

In one clinical trial study on insomnia conducted by Gula et al., Mongolian warm acupuncture significantly improved the quality of sleep by decreasing the awakening times and the sleep hours in the rapid eye movement (REM) period. The Pittsburgh sleep quality index (PSQI) and polysomnography indexes (PSGs) have been measured in Mongolian warm acupuncture clinical trials. The index of sleep quality, time for falling asleep, sleep hours, sleep efficiency, sleep disorders, and daytime functions in the Mongolian warm acupuncture group all improved significantly compared with those in the estazolam tranquilizer group [13].

Mongolian warm acupuncture may improve insomnia symptoms by upregulating the expression of certain genes in the hypothalamus. In the *p*-chlorophenylalanine (PCPA)-induced rat model of insomnia, the transcription levels of *Egr1, Btg2*, and *BDNF* were upregulated in the Mongolian warm acupuncture-treated group [63].

As proteins are executors of biological functions, it is necessary to identify the protein expression levels, modifications, and interactions in the Mongolian warm acupuncture-treated insomnia group. As reported by Gula A et al., in the hypothalamus of a rat insomnia model, 36 proteins showed increased levels and 45 proteins showed decreased levels in the insomnia model group compared with the healthy control. Twenty-eight proteins showed increased levels, while 17 proteins showed decreased levels in the Mongolian warm acupuncture-treated insomnia group compared with the insomnia model. The Mongolian warm acupuncture-treated insomnia group showed obvious recovery in protein expression levels and insomnia symptoms. Mongolian warm acupuncture may upregulate neuroactive ligand-receptor expression and oxytocin signaling to recover insomnia [64]. Another group also confirmed that warm acupuncture could improve sleep quality of patients with insomnia by promoting the expression of brain neurotransmitters (5-HT and GABA/Glu) and reducing that of norepinephrine (NE) [65].

Gula and colleagues showed that 141 miRNAs were changed when treated with Mongolian warm acupuncture in a rat insomnia model. The upregulation of miR-101a expression has been demonstrated to be directly associated with PAX8 regulation in rats treated with Mongolian warm acupuncture. Additionally, the levels of noradrenaline, dopamine, and glutamic acid and the interleukins IL-1, IL-2, and TNFα were decreased significantly in rats treated with Mongolian warm acupuncture [66].

#### Effects on neuropathic pain

Neuropathic pain is complex and chronic and is caused by damaged nerves. Acupuncture can relieve pain with few side effects and improve the quality of life of patients with neuropathic pain. In a double-blinded clinical trial, acupuncture relieved the pain of idiopathic trigeminal neuralgia. The mechanical thresholds were decreased, and the deep pain thresholds were increased in the acupuncture group. There was a reduction in secondary myofascial pain and mandibular limitations in the acupuncture group, and the changes could be maintained for more than six months [67]. In a clinical trial of angioneurotic headache patients, the efficacy index in headaches of the Mongolian warm acupuncture treatment group was superior to the efficacy index in the control groups. The total effectiveness rate was 90% in the research of Xiuping Bao [68]. As Li et al. reported Mongolian warm acupuncture could induce the recovery of idiopathic trigeminal neuralgia in the clinical observation of 80 patients, and the total effectiveness rate was 92.5% [69].

#### Effects on stroke sequelae

Stroke is emerging as a worldwide health issue threatening human health. Spastic paralysis after stroke (SPAS) is a common sequela of stroke and has received wide attention. The common treatment methods for SPAS are medications, surgical interventions, and physical therapy. However, these methods present disadvantages, such as the side effects of drugs, the invasive nature of the surgery, and the high cost of treatment.

According to two clinical studies by Zhang et al., acupuncture can significantly improve cerebral palsy motor and comprehensive functions in children, and the effect of acupuncture combined with rehabilitation is evidently superior to the effect of simple rehabilitation training therapy alone [70,71]. Mongolian warm acupuncture can increase recovery by enhancing immunity, regulating blood circulation, relieving pain, and relaxing muscles. In a clinical study, 70 patients with hemiplegic sequelae of stroke were treated with Mongolian acupuncture. After treatment, the Fugl-Meyer and Barthel assessment scores were significantly higher than the comparable scores of the control group.

Mongolian warm acupuncture can significantly improve the clinical conditions of patients with hemiplegia stroke sequelae and has positive significance in ensuring patient quality of life along with physical and mental health [72]. In another study, 60 patients with sequelae of cerebral infarction were subjected to Mongolian warm acupuncture. After a course of treatment, of the 60 patients, 3 were cured, accounting for 5.00% of cases, in 15 the treatment was markedly effective, accounting for 25.00% of cases, in 40 it was effective, accounting for 66.67% of cases, and in two cases it was ineffective, accounting for 3.33% of cases [73]. In Wang et al.’s clinical observations of 120 patients with sequelae of cerebral infarction, the total effectiveness rate of treatment in the Mongolian warm acupuncture group was significantly higher than the total effectiveness rate of treatment in the control group. After treatment, the ADL and FMA scores of the experimental group were higher than those of the control group, while the NIHSS score was lower than that of the control group [74].

### Other diseases

The main physiological characteristics of the elderly are the decline of the internal organs, the gradual depletion of essence and blood, the gradual loss of spirit, and the imbalance in Yin and Yang. However, multiple organ damage and multiple diseases often appear together and are the main pathological features in the elderly [75].

Mongolian warm acupuncture showed good therapeutic effects on lung function in elderly patients with stable chronic obstructive pulmonary disease. Through acupuncture and moxibustion, the main acupoints such as Beishu, Fengmen, Tanzhong, Dingchuan, Shenshu, and Zusanli were selected as matching acupoints for Xuehai, Fenglong, Chize, and Lieque. Mongolian warm acupuncture can regulate lung function, replenish lung Qi, remove wind pathogens, enhance Qi intake, and replenish vitality [76,77].

Diabetic peripheral neuropathy is a common chronic complication of diabetes, and its cause is mainly related to long-term high blood sugar levels, which lead to circulatory disturbances in nerve tissue metabolism, which in turn lead to peripheral neuropathy. At present, its pathogenesis is still in the research stage, and there is no clinical treatment for this disease. Studies have shown that Mongolian medicine warm acupuncture at the Zusanli, Sanyinjiao, Taixi, Waiguan, Shenshu, Pishu, Huantiao, Quchi, and Hegu acupoints can promote the smooth flow of the meridians and collaterals of the patient and increase the benign stimulation of the patient’s body and limb blood circulation. The warm Yang medicinal power of wormwood can dredge collaterals and disperse silt, promote local tissue nutrition and the improvement of symptoms in patients [78].

Prostatitis is a common high-incidence disease that mainly affects the population under 50 years of age. The reason the patient has related diseases is mainly because the pathogen invades the patient's prostate through urine, causing the patient to develop an infection. Under normal circumstances, the treatment of prostatitis patients is performed with Western medicine. However, the effects of using antibiotics for treatment are not obvious, and at the same time, the use of antibiotics can cause complications that may have a certain impact on the patient’s quality of life. Studies have shown that Mongolian warm acupuncture has a good effect on prostatitis, using mainly the Jingfuqian, Bladder, Wrist Acupoints, or 23 Zhui, Shen, and Elbow Neiwen acupoints for treatment [79].

Ischemic cerebrovascular disease has a high incidence and seriously threatens human health, being one of the three major causes of death. The mechanisms by which ischemia damages and causes the death of brain nerve cells are diverse and complex. Prevention and treatment of ischemic cerebrovascular disease is an urgent problem that needs to be solved. Mongolian warm acupuncture dealing with Dinghui and Heyi acupoints was found to significantly counteract the generation of free radicals in rats after ischemia, improve the body's ability to scavenge free radicals, and enhance SOD activity, which will help improve neurological deficits after stroke occurrence [80][8].

## Conclusions

Mongolian warm acupuncture is a kind of acupuncture that is still popular in the Inner Mongolia Autonomous Region of China and Mongolia. The tail of the needle used in Mongolian warm acupuncture is wrapped with moxa and burned (Fig. 2B) or, in nowadays, heated by electronics (Fig. 2C). Whether the needle tip temperature influences the therapeutic effect of acupuncture on disease is still unclear and needs to be further studied. At the same time, certain acupoints in TMM co-localize with those in TCM. Most acupoints in TMM include the brain and spinal cord, which are part of the “White Meridian” system. It has been proved that stimulating these acupoints can treat certain diseases in TMM, but the mechanisms still need to be further elucidated. With the development of technology and the standardization of scientific research, the physiological basis for the effect of Mongolian warm acupuncture will be better understood, contributing to the promotion of the therapy.
